# A Rape on Science

**Published:** 1887-07

**Authors:** 


### A RAPE ON SCIENCE.

The appearance in the columns of one of our most chaste and dignified exchanges, of a certain article on menstruation, by a "Female Medical Student," is as much a surprise as it is to be regretted. The subject matter is, of course, perfectly legitimate; but when the writer so far misconceives the province of a medical author, and so takes the name of "science" in vain, as to enter into a prurient discussion of the *sensations* attending the physiological process of ovulation, and of the sexual congress, giving, as she calls it, her own "sexual education," we think it time for a long-suffering reading public to protest. It is positively disgusting, and would shame the admirers of the Paul de Koek style, or put to blush the "Mysteries of a French Bedstead." A writer in the *Southern Practitioner*, for July, has been applauded for his criticism of the article; but we fail to see wherein he has been more modest than the "Female Medical Student," unless he be entitled to credit for plain language, where she has used the most lascivious inuendo, suggesting unchaste thoughts to even the coldest reader. Enterprise in medical journalism is to be commended, as in any other business; but when it takes the shape of vulgar revelations of sexual experiences by a shameless woman, who says, herself, that "under an incog. she strips off the last garment" (of decency) and "stands exposed in all her" naked vulgarity—hisses, and not applause, should greet both contributor and publisher. The idea of comparing the holy emotion of love to the *rut* of the brutes, the "season" of cattle, and the "*heat*" of the *bitch*, and that, too, in the name of "*science!*" is enough to excite sentiments of disgust and indignation. Out on all such pruriency and downright vulgarity. Love, the inspiration to the most noble deeds of which man is capable—the tie that binds society together, and on which the institution of marriage—holy wedlock is based—the mainspring of poetic fervor and impassionate eloquence, is higher, holier, purer than that instinct which man shares in common with the brute.

